# Report of Cases of Phthisis, Scrofula, and Other Diseases, Treated by the Chlorate of Potash

**Published:** 1861-09

**Authors:** E. G. Fountain

**Affiliations:** Davenport, Iowa


### ARTICLE II.

*Report of Cases of Phthisis, Scrofula, and other Diseases treated by the Chlorate of Potash, with Remarks on its Mode of Administration and the Importance of Using a Preparation free from Impurity.* By E. J. Fountain, A. M., M. D., of Davenport, Iowa.

Believing that all the unpleasant effects which some have observed as resulting from the administration of the chlorate of potash, and much of the loss of confidence recently expressed in its virtue as a remedial agent, to be due chiefly to the common use of preparations which are not perfectly pure, and, in some degree, to an injudicious method of administration, I feel it to be a duty incumbent upon me to offer some evidence in support of this belief, and in connection therewith, to illustrate the utility of the preparation by a report of some cases of recent date. I do this with reluctance, from the fact that I have already placed before the profession my views of some of the properties of this salt; and am well satisfied that its value will ultimately be determined by far more able observers, and to them and the profession at large I would willingly leave the further investigation of the subject. But, having presumed to suggest its use in what I supposed to be a new and untried field, and on theoretical grounds which have been considered untenable by excellent authority, (Dr. Squibb,) I cannot hesitate in again contributing something which I hope may aid at least in securing for it a fair trial. And this I will do, not in the spirit of hobbyism, to sustain opinions previously promulgated, but simply as an expression of facts which have some value, although too limited—as the experience of but one person—to be in all respects conclusive in character.

By a report from the Buffalo General Hospital, published in a recent number (December) of the American Medical Monthly, it appers that in three cases where it was administered, "the appetite was diminished, the diarrhœa and an anæmia exacerbated, and nausea produced or increased."—Without any special reference to its value as a remedy in phthisis, I desire to submit evidence of our ability to use it freely and under all circumstances where it is indicated, without producing any such effects upon the digestive organs; and not only with no such unpleasant effects, but with decided improvement to the appetite, an increase of strength, and recovery from anæmia, where previously existing.

I will preface the history of some cases by saying, that since I have been using the French chlorate of potash of absolute purity, I have not known of a single instance where it has produced either diarrhœa or loss of appetite. The difference in effects between this and the kind ordinarily kept by druggists will be apparent from some of the cases I am about to relate. These will be given mostly in outline for brevity, omitting much of detail which would be essential in a more formal report.

Since the publication of the paper which I had the honor of reading before the American Medical Association, I have

3

not had an opportunity of testing the treatment for incipient phthisis in any case of well-marked character; but a few cases of the disease in advanced stages will be related, for the purpose of showing some of the physiological and therapeutic effects of the remedy, and the extent to which it can be administered with safety and benefit.

*Case* 1.—Wife of the Rev. Mr. J., aged 30, of delicate form and a remarkably mild and amiable disposition; predisposed to consumption from her parents; mother of four children, who manifest in general appearance a strumous diathesis.

Began coughing in November, 1858, after an attack of bilious fever. Had hæmorrhage from the lungs three times in June of 1859, and slight symptoms of the same quite frequently since.

January 2d, 1860.—All the symptoms of advanced phthisis, well-marked: cough, purulent expectoration, emaciation, hectic, night sweats, &c. Physical signs plainly indicative of tubercles in both lungs, with cavities in the left. Cod-liver oil, quinia, anodyne expectorants, &c., were prescribed. The oil could not be tolerated by the stomach, and in a few days so great was the dyspnœa, that I substituted the chlorate of potash in drachm doses, three times daily. This produced no disturbance of the stomach, but an increase of appetite, diminution of expectoration, relief of the oppression and dyspnœa, and general improvement in all respects. After a short time, all other tonics were discontinued, the patient expressing a confidence in the belief that this alone was productive of any benefit. On two occasions the chlorate was omitted for a few days, from neglect or inability to renew the supply; and on each she rapidly failed in strength, and dyspnœa increased, with an aggravation of all unpleasant symptoms, which soon subsided again on resuming the use of the chlorate as before. With these interruptions, it was taken, three drachms daily, for a little more than two months; at no time producing either diarrhœa, nausea, or a sense of loathing. On the contrary, she regarded it as her only hold on life—taking it always without dislike, and frequently saying she knew she could not live without it. Other remedies of a palliative character were given as occasion required. Early in March she felt so much improved that she ventured on taking a walk, and imprudently took a very long one, requiring the ascent of a hill on returning. This greatly overtasked her strength, and from the extreme prostration which followed she never entirely recovered. A few weeks later I found her suddenly failing, with greatly increased dyspnœa. She directed my attention to the chlorate, saying the last package which she had obtained, appeared quite different from any which she had previously taken; having a *nauseous* taste, offended the stomach, and appeared to do her no good. After several attempts she was compelled to abandon it. I recognized at once an impure article, and returning it to the druggist, I was informed that his own supply had failed, and this he had obtained from another, knowing it to be inferior in quality, but the only kind he could find in the city. As he was in daily expectation of receiving a supply of the pure chlorate, I concluded I would try to do the best I could with my patient without any until it might arrive. In this expectation we were disappointed, and in the mean time it soon became apparent that life could be prolonged but a short time, and no further effort was made but to palliate her suffering and smooth her pathway to the grave. She gradually sank, and died on the tenth day from the discontinuance of the chlorate.

The following was my method of prescribing it: ℞.—Potass. chlorat. pulv., ℥ vj., Ft. chart. no. xvi. One powder to be taken each day, in three doses—each extemporaneously dissolved in a small quantity of hot water, sufficient to effect a solution; the first dose in the morning not to be taken on an empty stomach, but after breakfast. In this manner it was taken for more than two months, promoting sleep, improving the appetite, relieving the dyspnœa and pectoral distress, reducing the expectoration, and sustaining the strength of the patient.

*Case* 2.—Wm. P. aged 22, of consumptive predisposition by birth. In May, 1860, after being much reduced by chills and fever, he was suddenly attacked with profuse pulmonary hæmorrhage, and from that time had been gradually failing, with symptoms of consumption. Came from Ohio to be placed under my care early in October. The following are my notes of his condition when first examined October 12th: "General appearance indicative of emaciation and impoverished sanguineous system. Respiration hurried, and attended with dyspnœa on the slightest exertion. Cough frequent, with considerable expectoration. *No appetite*, frequent night-sweats. Debility so great that he requires assistance to go up stairs. Body greatly emaciated; ribs much depressed under the clavicles; marked dulness on percussion over all the left lung, anteriorly and posteriorly; slight degree of dulness over the superior portion of the right. No respiratory murmur audible beneath the clavicle on the left side, but loud bronchial respiration and broncophony, with a click attending each respiration. Lower down, slight indications of moist crepitation. Rales loud and abundant posteriorly, near the angle of the scapula. Dulness very marked in this region. On the right side, under the clavicle, the breathing coarse and insufficient, with prolonged expiratory murmur."

*Treatment.*—A wine-glassful (℥ ij.) of the saturated solution of the chlorate of potash, (one drachm of the salt,) three times a day, after meals, and as much exercise in the open air as strength will permit. Rapid improvement soon followed this treatment. In a few weeks his strength improved so that he could take quite a long walk each day, ascend stairs without assistance, and eat heartily with a *good appetite*. Cough and expectoration very much diminished. Patient feels like another person, and is in all respects very much improved. The chlorate is taken three, and sometimes four times a day, without reluctance or any inconvenience. Lungs re-examined November 4th. *Right side*—no dullness on percussion; respiratory murmur clear and well evolved. *Left side*—dullness the same as when first examined; the same bronchial sounds, with the click; but an absence of rales where they were before abundant.

A few days after this he was influenced, contrary to my wishes, to visit some relatives in St. Paul, with the intention of remaing through winter in that country. As I apprehended, he contracted a cold on the boat, which he further aggravated by undue amount of exercise and great imprudence immediately after his arrival. As I have been informed by his physician in St. Paul, symptoms of great prostration ensued under which he rapidly sank, and died within a few days after his arrival. The points of interest in this case are the rapid improvement following the use of the chlorate, with a corresponding change in his physical symptoms revealed by auscultation; a perfect tolerance of the remedy, which never produced nausea or diarrhœa, but greatly increased his appetite and power of digestion.

*Case* 3.—Miss Anna H., aged 17—had whooping-cough last fall, followed in the winter by a cough, which her family believed to be indicative of consumption. During this time she was attended by a homœpath, who was discharged on account of his inability to relieve her of a more recent and severe attack of illness. I was called to see her June 22d, when she was supposed to be in a hopeless condition. I found her laboring under a frequent and suppressed cough, with rapid and labored respiration, and frequent, but feeble, pulse—the latter 128, and the former 61 per minute. This great discrepancy between the pulse and respiration, by excess of the latter in frequency, indicated some serious impediment to the due performance of the function of respiration. Auscultation confirmed this opinion. Subcrepitant and mucous rales over all parts of the right lung, and a large portion of the left. Slight but not well-marked dullness over a part of the right. No expectoration. Diagnosis: capillary bronchitis, involving a general pulmonary congestion and imperfect aeration of the blood.

*Treatment.*—An ounce of the saturated solution of the chlorate of potash, every three hours. On the morning following, I found her much relieved. Respiration 44, pulse 96; increasing in the afternoon to 48 and 110. Ordered quinia, 10 grains for the next day, and five grains for each of the three days following. Chlorate continued the same. June 26th, respiration 24, pulse 82; very few rales to be heard. Steady improvement followed. The chlorate was continued, in gradually diminished doses, for several weeks; during which time she recovered from her illness, excepting the symptoms of an organic disease, disconnected from the pulmonary organs.

*Case* 4.—Mrs. B., aged 44, has for many years been in delicate health, but with no well-defined indication of organic disease. Is subject to some cough, and has several times had slight hæmoptysis. Has frequently taken cod-liver oil with benefit. In the spring of the present year, her debility was greater than usual; appetite very poor; nights passed restlessly, with but little sleep; sense of oppression and distress in the chest; cod-liver oil and ordinary tonics given, with no benefit, or but very little; substituted solution of the chlorate of potash, table-spoonful three times a day. This has been taken during all the past summer and fall, and is still being used, though not with such regularity at the present time, (Dec.) The lady often tells me that she cannot do without it. Her appetite and general strength have been greatly increased, and in every way she is much relieved and benefited by it. Whenever she omits taking the solution for a number of days, she tells me she always feels the want of it, and experiences immediate improvement upon resuming its use.

*Case* 5.—Rev. Mr. C., aged 26, applied to me in September for the relief of a chronic eruption of an eczematous character, for which I prescribed a wine-glassful of the saturated solution of the chlorate, morning and evening. A few weeks after this, he surprised me by saying that he felt himself being greatly relieved of a pulmonary difficulty, which had been gradually increasing upon him for several years; but he had said nothing about it when he applied to me, as he believed that no medicine could help him. This difficulty was a weakness of his lungs, attended with a slight cough; gradually increasing dyspnœa; difficulty and pain in expanding the chest; loss of strength in the voice; tenderness of the chest when pressed; no appetite, uneasy, restless nights, and general debility. Though attending to his duties as assistant pastor of a Catholic church, he felt unequal to their proper performance, and feared they would soon have to be abandoned. All this I knew nothing about when I gave him the Chlorate, nor did he suspect that it would have any influence over these symptoms; yet, on using it, he was soon surprised to find himself being greatly relieved of them all, and he came to inform me of the change in his condition: dyspœna relieved; cough almost gone; *appetite good;* color and appearance much improved. He is still taking it the same way, having now been using it for three months, and he looks and feels almost like another person. He has grown much stouter, has a healthy complexion, a strong voice, and excellent appetite. On several occasions he has omitted the chlorate for a time, and always found his old symptoms returning; and thus by his own intelligent observation, without any prompting from me, or any previous knowledge of the properties of the chlorate, he has been fully convinced of its efficacy in relieving him of his pulmonary difficulty and its value as a general tonic.

Although no examination of the lungs was made in this case, owing to the fact that no difficulty there was suspected, yet, from its peculiarity, it is of special interest and importance as a demonstration of the power of the chlorate over the organs of respiration, and its efficacy as a tonic. Of this there can be no mistake, as the result was quite as unexpected as the difficulties relieved were unknown to me, and there was, therefore, no possible chance for any self-deception, by reason of my partiality for the chlorate in such cases.

*Case* 6.—Miss Josephine C., aged 12, has had a fœtid discharge from one ear all the time, and frequently from both, since she was an infant eleven months old. During this period, she has had all manner of treatment from different medical attendants. Iron, iodine, cod-liver oil, iod. of potassium, &c., had been prescribed at different periods, and a great variety of local applications, all without any material benefit. The family have been living in good circumstances, and have made every possible effort for the relief of the patient. The hearing in one ear was much impaired, the discharge constant and very offensive. General state of health not good; anæmic, appetite poor, subject to headache almost constantly.

June 20th, 1860.—Upon being consulted by the mother, I concluded to give trial to the chlorate of potash, on the supposition that the disease was of a strumous character. I directed three table-spoonsful of the saturated solution to be taken morning and evening, with no other remedy and no local treatment, except occasionally cleansing the ear with warm water. In less than two weeks the mother called upon me to express her gratitude for the surprising change which the treatment had produced. The discharge had ceased entirely for the first time in eleven years, and she was looking and feeling in all respects much better. Condition four weeks later: No return of the discharge from the ear, and healthy wax is appearing for the first time since she was a child. Hearing improved. Headache has left her almost altogether; (before taking the Chlorate, it was the exception to the rule to be free from it;) appetite excellent; general health quite restored—better than she had ever enjoyed before—and she is surprising her parents and friends by the rapidity with which she is gaining flesh. In place of her former anæmic, waxy appearance, she is now fair and ruddy. In the six weeks that she has been taking the chlorate, (which had produced no inconvenience,) she has been perfectly cured of an extremely unpleasant difficulty which had existed from infancy, and changed from a delicate, unhealthy appearing girl, to a perfect picture of health and beauty.

*Case* 7.—Child of Mr. D., age 5; for a year or more had been troubled with an eruption all over the body; appeared first in small red points, soon progressing into a pustular eruption, and degenerating into dry scabs, which coalesce into patches of various size and irregular form, occasioning but little pain, but great annoyance from itching. Ordered Chl. Pot., a table-spoonful of saturated solution four times a day. In a few weeks the disease entirely disappeared. Previously she had suffered much with headaches and had a very offensive breath, from both of which she has been quite relieved while taking the chlorate.

*Case* 8.—Mr. Wm. M., a gentleman of good constitution and usually of good health. Applied for the relief of a painful, indolent-looking tumefaction on the leg, over the front of the tibia, in appearance threatening to form an open ulcer. Skin surrounding it for several inches of a dark, livid color. Cause unknown, but from appearance believing it to be from some derangement of the blood, I prescribed the chlorate of potash, a wine-glassful of the sat. sol., morning and evening. The livid color soon began to disappear, and a gradual but steady restoration to a healthy condition followed. After he had been taking it a number of days, he remarked to me that the medicine had given him a *great appetite*, which previously was not good; a fact that I did not know until he voluntarily informed me of its marked improvement.

*Case* 9.—Mr. W., aged 35, I found very much reduced from profuse suppuration, proceeding from numerous and extensive ulcers and sinuses of a scrofulous character on the neck, originating in the chain of lymphatic glands. Had been under treatment for some months, the suppuration being constantly encouraged by poulticing. The sinuses were freely opened immediately, caustic and stimulant applications applied, and tonic treatment, including iron, quinia, iodine and cod-liver oil, prescribed in liberal quantity. Constitutional symptoms improved, and amount of suppuration much reduced; but a number of weeks I tried in vain to promote healthy granulation and cicatrization, until I gave him drachm doses of the Chlorate, three times a day. This soon changed the livid color to a healthy arterial hue, and from this time permanent improvement began and progressed, though slowly, until quite restored. For about two months he took three drachms of the chlorate daily, without diarrhœa or nausea. He took it in the form of a wine-glassful of the sat. sol. after each meal. It never produced nausea or diarrhœa, and he frequently said to me that it gave him a good appetite.

*Case* 10.—Daughter of Mr. S., age 2 years and 10 months, has been losing flesh and color for some weeks, with no well-defined symptoms of any disease that could be recognized. Iron and quinia failed to relieve the anæmia. Parents became much alarmed. Abscesses began to form on the fingers and elsewhere; slight scratches degenerated into ugly sores; the breath quite fœtid ; no appetite; body much emaciated. Ordered a dessert-spoonful of the saturated solution four times a day. Soon the little patient began to show signs of improvement, which steadily progressed to complete recovery in a few weeks.

*Case* 11.—A little girl aged 4 years, was brought to my office from the country, July 28th. For the past two years has had a constant and very fœtid discharge from the nares, acrid in character, excoriating the lip. Sometimes lumps of hard, dark-looking matter discharged. Health poor generally. Prescribed a table-spoonful of the saturated sol. of the chlorate, with a litfle comp. tinct. of bark, three times a day. August 11th, patient again brought to me by the mother. Change in condition very striking. Discharge almost entirely checked, and no offensive odor. The mother remarked, "She is like another child ; has a better appetite, and is much brighter." Ordered a continuation of the treatment, and have not seen her since.

*Case* 12.—Mrs. L., suffering with a large carbuncle on the back, and smaller ones appearing elsewhere on the body. Prescribed Chlorate of Potash, half an ounce daily. In a few days she informed me that it produced diarrhœa and nausea, and tasted repulsive. I examined the preparation, and at once recognized the common, impure chlorate. I immediately ordered some to be obtained from one of our best druggists, where they keep none but the French chlorate. This, she soon informed me, tasted different; was not nauseous, and produced no diarrhœa. The patient is now recovering on its use. The large carbuncle, after being opened, is discharging freely and looking well, and the smaller ones apparently subsiding. In this case, like the first, it will be observed that a marked difference was noticed by the patient in the effects of different samples of the chlorate.

*Case* 13.—*Morbus Coxarius.*—Eliza Bennett, aged 6. For two years previous to my attendance, has suffered with all the characteristic symptoms of tuberculosis of the hip-joint, which has been seen and treated at different times by a number of physicians. I was called to the case in June of the present year, (1860.) Found the child emaciated, attempting to hobble on a crutch; in appearance and expression the picture of distress and long suffering. Inversion and apparent shortening of the limb; tissue about the joint hardened and prominent, through which there were five openings from the joint, discharging, more or less, at the time of my visit. There was also an opening over the lower point of the sacrum, from which two or three small pieces of bone had been discharged.

I could hope to do but little for the case, as the family lived in the country, in a miserable shanty, with no ability to take proper care of the child. As I had commenced testing the chlorote of potash in all forms of scrofulous affections, I prescribed it in this case on general principles, and as a matter of experiment. I ordered a table-spoonful of the saturated solution, to be given three times a day.

I did not see or hear of the case again until two months after, when I was sent for to see the father, who was sick with fever. After talking with him for a few moments, the mother asked me to look at my little patient with the hip disease. To my astonishment, I found the child, at that moment, bringing in an armful of wood—a bright, healthy-looking little girl, walking erect, without any apparent lameness. I could hardly believe it was the same child until I examined the hip and found all the marks of the old openings now perfectly closed. She had taken the chlorate regularly, improving constantly and rapidly under its use. The discharge from the openings gradually ceased, the appetite and strength improved, and the stiffness of the joints relaxed, until, within two months, it could be used almost as freely as the other. Careful measurement showed there was no real shortening of the limb. By watching carefully, a very slight lameness can be detected in walking. In October I exhibited the child before the Scott County Medical Society, some of the members of which had seen my patient before I commenced my treatment, and knew from their own observation the full extent to which the disease had progressed. By this time she was as healthy looking as any little girl that could be found—plump and rosy-cheeked, with a cheerful, happy expression.

At this meeting all the members examined the case, and are witnesses to a complete recovery of a bad case of morbus coxarius under the influence of the Chlorate of Potash.

I might add other cases to the above list, in testimony of the value of the Chlorate of Potash, in various affections, but these are sufficient for my present purpose. I will simply add, that in the practice of my friend, Dr. J. M. Adler, of this city, the use of the Chlorate of Potash is attended with equally favorable results. He informs me that in two cases of incipient phthisis, the treatment has been successful in arresting the disease and restoring the patient to sound health. But these and other cases, which I may obtain elsewhere, will be reserved for some future occasion. Such as I have here reported will, I trust, be sufficient at least to encourage others to give the remedy a fair trial. In this connection, and for the purpose just expressed, I will copy the following, from a communication to the *American Medical Times*, of December 15th, 1860, from T. C. Moffatt, M. D., of the Seamen's Retreat, Staten Island.

"My experiments in the use of the chlorate of potash in phthisis, as employed by Dr. Fountain, of Iowa, have not yet been of sufficient duration to warrant me in speaking of it with very great confidence; but I am encouraged to persevere in the use of it more and more daily. One patient in the advanced stages of the disease in question, has been using it in one-half ounce doses, daily, for eight days. Before commencing this treatment his breathing was difficult and hurried upon the slightest exertion; his lips were livid, and extremities cold. He was able to get but little sleep, owing to an almost constant cough; and his appetite never good, was sometimes so poor that he could take no nourishment at all for an entire day. His general appearance now strongly confirms the testimony which he gives, that he sleeps nearly all night undisturbed. The pain and constriction of the chest are much relieved; and expectoration, formerly quite profuse, has ceased almost entirely. His condition in every respect is materially improved. Two other patients, also in advanced phthisis, having been using the article but three or four days. One of them speaks confidently of decided improvement, and says that he breathes freer, and sleeps and eats better. None of them complain, as yet, of any inconvenience whatever from the use of it. I hope to be able to test the efficacy of this article in the incipient stages of the terrible scourge in question, which swells our mortality list to nearly one-half, and everywhere is proverbially destructive among the men of the sea."

Inasmuch as the Chlorate of Potash must be given in large doses, and in all chronic cases continued for a long time, in order to derive full benefit from its use, it is of the utmost importance that the article should be perfectly pure. I wish to impress this upon the minds of all who use it, that it may not be unjustly condemed for what may often be the effects of impurity in the preparation. I do not wish to be understood as saying that none but the French Chlorate is perfectly pure, but only that I am acquainted with no other that I can depend upon, and that such as druggists usually keep is inferior to it. Physicians must look to the quality of the article with which their patients are furnished, or they are likely to be deceived, and unjustly to condemn it. For the benefit of the readers of this journal who live at a distance, and in reply to inquiries which have been made of me by letters, I will here say, that none better can be obtained or desired than the French Chlorate of Potash which is furnished by the house of Dazell, Marsh & Gardiner, No. 10 Gold street, New York. It is in small scales and flakes, of brilliant appearance and pearly whiteness; while the ordinary variety is in larger crystals and lumps, and not so bright in lustre. When pulverized, the difference in appearance is still apparent, the latter being duller and darker; and it is also more difficult to reduce to a fine powder. In its administration, I do not now give it as I did in the cases reported in my paper on phthisis, dissolved in an unnessarily large quantity of water, but generally make a saturated solution with hot water. As it cools some will, of course, be precipitated, but the water is thus, and thus only, sure to be saturated. In cold weather I direct this to be kept in a warm room, that the temperature of the liquid may not fall too low, and thereby precipitate more of the chlorate. At summer heat it will retain about an ounce to a pint, making fifteen grains to a table-spoonful, and one drachm to a wine-glassful, (2 oz.,) my ordinary dose for an adult. This I give from one to four times a day, as circumstances require, and usually after meals. I have one patient who prefers taking the dry powder in in his mouth and washing it down with a drink of water, and I am not sure but I would prefer taking it this way myself.

Believing the chlorate of potash to be a *tonic*, *alterative* and *blood depurant* superior, in many respects, to anything in our materia medica, I do not hesitate again to urge its claims upon the attention of the profession. From the time when, in 1851, I discovered its utility as a remedy for mercurial ptyalism, I have been testing its properties in many directions, and with constantly increasing confidence in its virtues, and (in the language of Dr. Hutchinson) "its scarcely less than wonderful power." I believe there is no tonic and alterative comparable to it in all manner of scrofulous diseases, and every form of tuberculosis, in the seqelæ of the exanthemata, in adynamic fevers, and asthenic types of disease with depressed vital power, in every condition involving an imperfect æration of the blood, and many derangements of the system resulting in abscesses, eruptions, ulcers, &c. In my opinion, it has this wide range in its application fly reason of its *oxydizing* properties, in support of which I may have something to say hereafter.—*Am. Med. Monthly.*

